# A Lipidomic Analysis of Placenta in Preeclampsia: Evidence for Lipid Storage

**DOI:** 10.1371/journal.pone.0163972

**Published:** 2016-09-29

**Authors:** Simon H. J. Brown, Samuel R. Eather, Dilys J. Freeman, Barbara J. Meyer, Todd W. Mitchell

**Affiliations:** 1 School of Biology, University of Wollongong, Wollongong, NSW, Australia; 2 Illawarra Health and Medical Research Institute, University of Wollongong, Wollongong, NSW, Australia; 3 School of Medicine, University of Wollongong, Wollongong, NSW, Australia; 4 Institute of Cardiovascular and Medical Sciences, University of Glasgow, Glasgow, United Kingdom; Faculty of Animal Sciences and Food Engineering, University of São Paulo, BRAZIL

## Abstract

In preeclampsia, maternal insulin resistance leads to defective expansion of adipocytes, enhanced adipocyte lipolysis, up-regulation of very low density lipoprotein synthesis, maternal hypertriglyceridaemia and the potential for ectopic fat storage. Our aim was to quantitate and compare the total amount and type of lipid in placenta from pregnancies complicated with preeclampsia and healthy pregnancies. Quantitative lipid analysis of lipid extracts from full thickness placental biopsies was carried out by shotgun lipidomics. Placental lipid profiles from pregnancies complicated by preeclampsia (n = 23) were compared to healthy pregnancies (n = 68), and placenta from intrauterine growth restriction pregnancies (n = 10) were used to control for gross differences in placental pathology. Placentae from pregnancies complicated with preeclampsia had higher neutral lipid content than healthy placentae (40% higher triacyglycerol (P = 0.001) and 33% higher cholesteryl ester (P = 0.004)) that was specific to preeclampsia and independent of maternal gestation.

## Introduction

Preeclampsia (PET) is a leading cause of pregnancy-related maternal and offspring mortality and morbidity. It occurs in 2–10% of pregnancies and is unique to humans [1]. PET is a multi-factorial disease with a number of presenting phenotypes caused by a primary defect in trophoblast invasion followed by an atypical maternal vascular response resulting in hypertension and proteinuria [1, 2]. Maternal obesity is a risk factor for PET [3] and an abnormal maternal metabolic adaptation to pregnancy, reflective of metabolic syndrome, is a key feature of the disease [4].

Maternal gestational hyperlipidemia is physiological and provides for the energy demands of the fetus as well as supplying lipid precursors necessary for fetal development [5, 6]. In healthy pregnancy, mothers store fatty acids in adipose tissue and, as a consequence of the action of pregnancy hormones, maternal insulin resistance develops in mid to late gestation [7]. This leads to increased adipocyte lipolysis, up-regulation of very low density lipoprotein (VLDL) synthesis by the liver and gestational hypertriglyceridaemia [8]. We have previously shown that in PET, there is evidence that mothers are less able to expand their adipose tissue and their adipocytes are more insulin resistant resulting in increased lipolysis[9]. Excessive lipolysis and reduced capacity to store fatty acids in adipose tissue, such as is seen in type 2 diabetes, can lead to ectopic fat accumulation in the liver and other tissues with downstream pathological consequences resulting from lipotoxicity [10]. The consequences of inappropriate fatty acid handling in pregnancy can include vascular dysfunction [4, 11], increased insulin resistance [12] and defects of long chain polyunsaturated fatty acid (PUFA) mobilization [13]. It is not known to what extent ectopic fat accumulation occurs in PET.

In pregnancy, the placenta is an additional potential site for fat storage. Indeed, lipid storage droplets in placenta have been observed in a rodent model of PET using histochemical staining [14]. In humans, higher phospholipid (PL) and cholesterol content of placental tissue and the decidua basalis have been observed in PET using enzymatic detection [15, 16], as have differences in lipid mass spectral ion intensity between placentae from healthy and PET pregnancies [17]. Nevertheless, there are no quantitative data regarding neutral lipid storage in human placenta and there has been no quantitative analysis of placental lipid profile. The aim of this study was to compare total lipid content, neutral lipid content, and also the lipid profile of placentae from women with healthy pregnancies with those from women with PET. Placentae from pregnancies complicated by intrauterine growth restriction (IUGR) were also analyzed to control for the effects of gross placental pathology on lipid composition. This study successfully achieved the aim to comprehensively assess the lipid content placentae in both healthy and complicated pregnancy.

## Methods

### Sample collection and preparation

Women with PET (n = 23), with IUGR (n = 10) and controls with uncomplicated pregnancies (n = 68) in the third trimester of pregnancy were recruited from the Princess Royal Maternity Hospital, Glasgow. The study was approved by the Glasgow Royal Infirmary Local Research Ethics Committee and women gave written informed consent. PET was defined according to the ISSHP criteria (diastolic blood pressure >110 mmHg on one occasion, or >90 mmHg on repeated readings, with proteinuria of ≥ 0.3g/24 h, or 2+ on dipstick testing, in the absence of renal disease or infection). IUGR was defined as an estimated fetal weight <5^th^ percentile for gestation with associated oligohydramnios (amniotic fluid index <5) and/or abnormal umbilical artery blood flow on Doppler ultrasound. None of the women had a medical history of metabolic disease or had suspected fetal anomalies likely to contribute to reduced fetal growth. Subject characteristics were recorded at time of sampling. Delivery details were recorded from patient notes. Customised birth weight centiles were calculated using the Gestation Network Centile Calculator 5.4 (http://www.gestation.net/birthweight_centiles/centile_online.htm). Placental biopsies were collected at delivery. Biopsies were flash frozen in liquid nitrogen and stored at -80°C until analyzed.

### Blood Pressure and Urinary Protein Measurement

Blood pressure measurements were taken at all routine antenatal visits by a midwife using an automated sphygmomanometer (A&D digital BP machine) in the left arm in a seated position. Urinalysis was initially screened using a combiscreen dipstick (Analyticon Biotechnologies) and, if abnormal on visual screening, confirmed on dipstick reader (Combiscan 100, Analyticon Biotechnologies). For women recruited just prior to undergoing Caesarean section, pre-operative blood pressure was taken using a Dinamap^®^ (GE Healthcare) in the left arm with women in a semi-recumbent position and repeat urinalysis was not performed if a recent measurement in clinic had been negative. In PE women, third trimester blood pressure measurements were taken in the left arm in semi-recumbent position using a Dinamap^®^.

### Chemicals

All solvents were high-performance liquid-chromatography (HPLC) grade or liquid-chromatography mass-spectrometry (LC-MS) grade when available. Methyl-*tert* butyl ether (MTBE) was sourced from Sigma (Castle Hill, NSW, Australia) and methanol and chloroform were obtained from VWR (Murarrie, QLD, Australia). Ammonium acetate was LC-MS grade (Fluka, Castle Hill, NSW, Australia).

### Lipid extraction

Lipid extraction was performed according to the modification of the method of Tran *et al* [18]. In brief, 10–30 mg of frozen placental tissue were homogenised in 300 μL methanol using a bead homogeniser (FastPrep-24, MP Biomedical, Seven Hills, NSW, Australia). The homogenate (240 μL) was added to 800 μL MTBE and 50 μL of methanol containing standards listed in Table A in S1 File. Samples were vortexed overnight at 4°C. Ammonium acetate (200 μL of 150 mM) was added to induce phase separation. The upper organic layer was removed to a new vial, dried under a stream of nitrogen at 37°C, resuspended in methanol:chloroform (2:1 v/v) (100 μL per 10 mg tissue) and stored at -20°C until analysis. Extracts were diluted in 30-fold into methanol:chloroform (2:1 v/v) containing 5 mM ammonium acetate prior to mass spectrometric analysis. All samples subjected to ESI-MS were at a concentration below 20 μM, conditions at which ion-suppression effects are minimal [19].

### Mass Spectrometry

Mass spectra were acquired using a chip based nano-electrospray ionization source (TriVersa Nanomate^®^, Advion, Ithaca, NY, USA) coupled to a hybrid linear ion trap-triple quadrupole mass spectrometer (MS) (QTRAP^®^ 5500, ABSCIEX, Foster City, CA, USA) according to the methods of Tran *et al. [18]* Target lipids and MS aquisition parameters are shown in Table B in S1 File. An automated method was utilized to cycle all experiments for the duration of each polarity. Mass spectra were acquired at 200 *m/z* units.s-1 and averaged over 80 cycles. Each sample was measured once. Data were analyzed with LipidView^®^ (ABSCIEX) software version 1.2, including smoothing, identification, removal of isotope contribution from lower mass species, and correction for isotope distribution. Ionized lipids detected within a mass tolerance of 0.4 *m/z* units with a signal-to-noise ratio (s/n) over 10 were included in the analysis.

Quantification of lipid species was achieved by comparison of the peak area of individual lipids to their class-specific internal standards after isotope correction. For TAG class lipids, the signal for all fatty acids at a given mass was summed and the sum-composition TAG species (e.g. TAG 48:3) are reported. The fatty acids detected for each sum-composition TAG species are shown in Table C in S1 File. In the case of phospholipids (PL), to obtain quantification of the molecular species of phosphatidylcholine (PC), phosphatidylethanolamine (PE) and phosphatidylserine (PS), firstly quantification of sum lipid species (headgroup plus total fatty acid carbon number and double bond number, e.g. PC 34:1) was obtained by comparison to internal standards in head-group scans in positive mode. Next, negative ion mode precursor ion scanning for fatty acyl chains was used to determine respective fatty acyl chains for each lipid species. Ions detected at masses that could be assigned to ether-linked or odd-chain fatty acids were assumed to be ether-linked species. In cases where more than one isobaric molecular species was detected in a class by negative fatty acyl scans, the fractional intensities of each isobaric species were applied to the quantification obtained in positive mode head group scans. Lipid classes were determined as the sum total of all molecular lipid species in each class.

### Statistics

Data were tested for normality with the Shapiro-Wilk test prior to statistical analysis. Triacylglycerol (TAG), ceramide (Cer) and lipid class data were log-transformed to achieve normality prior to statistical analysis. Patient characteristics are shown as mean (standard deviations [SD]) for continuous variables and number (%) for categorical variables. Total levels of the various lipid classes and species are presented as mean ± standard error of the mean (SEM). PET, IUGR and healthy control group means were compared using a one-way ANOVA, with P<0.05 considered significant for demographic and lipid class data and P<0.01 considered significant for lipid molecular species data. In the event of a significant result a Tukey post-hoc test was performed to identify which groups were significantly different. Associations between variables were assessed using Pearson’s correlation analysis. The impact of potential confounding variables was assessed using general linear models. SPSS v21 (IBM) and Minitab Vs17 were used for statistical analysis.

## Results

### Maternal characteristics

Maternal characteristics are shown in Table 1. The PET group had higher (20%) systolic blood pressure (SBP) and higher (28%) diastolic blood pressure (DBP) than healthy controls. Gestational age at delivery was lower in PET (8%) and IUGR (8%). Birth weight centile (BWC) were lower in PET (60%) and IUGR (98%) pregnancies compared to controls. Elective Caesarean section rate was higher in the healthy pregnancy control group compared to PET.

**Table 1:** Maternal demographics and plasma lipids.

Variable	Control (n = 68)	PET (n = 23)	IUGR (n = 10)	*P*
Age (years)	30.4 (5.1)	29.5 (5.8)	29.7 (4.9)	0.83
BMI (kg/m^2^) *	28.9 (6.4)	30.1 (7.4)	24.7 (5.5)	0.068
Primiparous n (%)	27 (40)	14 (61)	4 (40)	0.20
Smoker n (%)	14 (21)	4 (17)	5 (50)	0.099
SBP (mmHg) *	121 (15)^1^	144 (25)^2^	113 (4)^1^	<0.0001
DBP (mmHg) *	71 (10)^1^	91 (16)^2^	72 (3)^1,2^	<0.0001
Gestational age at delivery (days)	275 (9)^1^	253 (21)^2^	253 (21)^2^	<0.0001
BWC	55 (31)^1^	22 (26)^2^	1 (2)^2^	<0.0001
Samples collected when not in labour (%)	51 (75)^1^	6 (26)^2^	6 (60)^1,2^	<0.0001
Fetal sex n (% male)	32 (49)	13 (57)	3 (33)	0.50

BMI, Body mass Index; SBP, systolic blood pressure; DBP, diastolic blood pressure; BWC, birth weight centile;

Values are mean (SD) for continuous variables and number (%) for categorical variables.

ANOVA was used to test for differences among groups (*on log transformed data). Different superscript numbers indicate differences between individual groups using post hoc Tukey test. Significance level P<0.05.

### Placental Lipid Quantification

Shown in Table 2 are the overall lipid class profiles in placental tissue from healthy control, and complicated pregnancies PET and IUGR. Quantification of lipid molecular species, as well as lipids that are different between control and PET, are reported in Table D in S1 File.

**Table 2:** Quantitative comparison of class total lipid profiles in placenta.

Lipid Class (μmol/g tissue)	Control (n = 68)	PET (n = 23)	IUGR (n = 10)	*P*
TAG*	0.20 (0.11)^1^	0.27 (0.12)^2^	0.15 (0.05)^1^	0.001
CE*	0.31 (0.10)^1^	0.42 (0.16)^2^	0.28 (0.10)^1^	0.004
Cholesterol	2.9 (0.5)^1^	3.4 (0.8)^2^	3.2 (0.7)^1,2^	0.006
PC	3.2 (0.8)^1^	3.7 (1.1)^2^	3.5 (1.0)^1,2^	0.025
PE	0.93 (0.25)	1.07 (0.38)	1.01 (0.31)	0.169
PS	0.36 (0.09)	0.40 (0.10)	0.40 (0.13)	0.127
SM	0.71 (0.18)	0.82 (0.26)	0.88 (0.22)	0.014
Total PL	5.2 (1.2)^1^	6.0 (1.7)^2^	5.7 (1.6)^1,2^	0.029
Cer	0.06 (0.02)	0.06 (0.03)	0.06 (0.02)	0.605
Total Lipid	8.6 (1.7)^1^	10.1 (2.4)^2^	9.4 (2.4) ^1,2^	0.006

CE, cholesteryl ester; Cer, ceramides; IUGR, intrauterine growth restriction; PC, phosphatidylcholine; PE, phosphatidylethanolamine; PET, preeclampsia; PL, phospholipid; PS, phosphatidylserine; SM, sphingomyelin; TAG, triacylglycerol.

Values are mean (SD).

ANOVA was used to test for differences among groups (*on log transformed data). Different superscript numbers indicate differences between individual groups using post hoc Tukey test. Significance level P<0.05.

PC is the most abundant lipid in placental tissue (~36% of total lipid) from healthy pregnancies, found at 3.2 ± 0.8 μmol/g. Cholesterol was nearly as abundant at 2.9 ± 0.5 μmol/g. Other PL classes were at substantially lower abundance, with PE, sphingomyelin (SM) and PS found at 0.93 ± 0.25 μmol/g, 0.71 ± 0.18 μmol/g and 0.36 ± 0.09 μmol/g respectively. Cholesteryl ester (CE) and TAG were similar in abundance at 0.31 ± 0.10 μmol/g and 0.20 ± 0.11 μmol/g respectively. Low levels of Cer were detected at 0.06 ± 0.02 μmol/g.

Both the neutral storage lipids, CE and TAG, were higher in placenta complicated with PET compared to healthy controls and IUGR. Both TAG and CE levels were substantially higher in placenta complicated with PET (40% and 33% respectively) versus healthy controls. These differences were retained after correcting for gestational age at delivery. Cholesterol (16%), PC (18%), and total lipid (18%) were also higher in placenta complicated with PET compared to healthy controls.

### Placental neutral storage lipids

**Fig 1:**
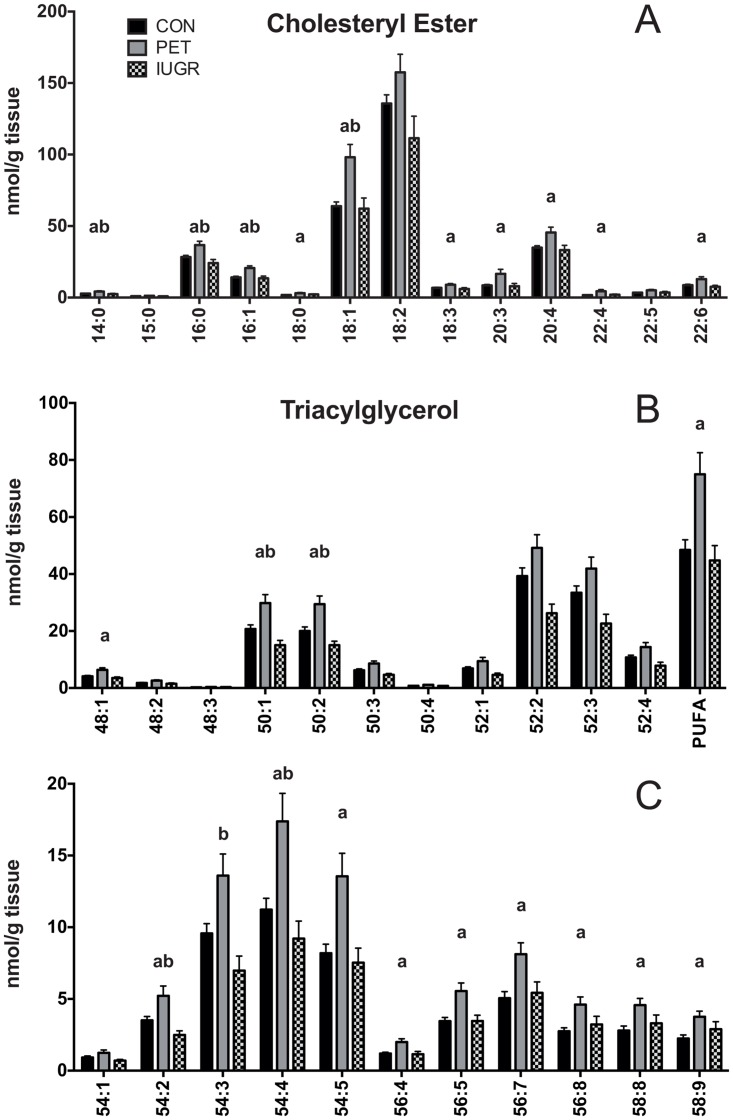
Storage Lipid Profiles in Placenta. Quantitative comparison of A) triacylglycerol and B) cholesteryl ester molecular lipid profiles in placenta between control (N = 68), PET (N = 23) and IUGR (N = 10). Values are shown as the mean of all measurements ± SEM. a = p < 0.01 versus control, b = p < 0.01 versus IUGR.

Molecular speciation of the neutral storage lipids CE and TAG was compared between groups (Fig 1). Thirteen CE species were quantified, with ester-linked carbon chain of lengths from C14 to C22, shown in Fig 1A. CE is dominated by unsaturated 18 carbon species, with CE 18:1 and 18:2 together representing 63% of total CE in healthy placenta. CE 20:4 and 16:0 are also abundant, at 11% and 9% respectively. All other species make up 5% or less of total CE. Ten of the 13 CE species were higher in concentration in PET compared to healthy controls, and three CE species (16:0, 16:1 and 18:1) were higher in comparison to both healthy and IUGR controls.

A total of 22 TAG lipid species are reported, shown in Fig 1B and 1C. The fatty acids detected in each TAG molecular species are shown in Table C in S1 File. The most abundant TAG species are TAG 52:2 and TAG 52:3, each representing nearly 20% of total TAG. TAG 50:1 and 50:2 are also abundant at 11% each of total TAG. Highly unsaturated TAG species with four or more double bonds were also detected across the entire range of carbon chain lengths, with these TAG species containing PUFAs 18:2, 20:4 or 22:6 (Table C in 1 File). Similar to CE, a widespread higher concentration of TAG molecular species was observed in placenta complicated with PET. Of the 22 TAG species detected, 13 were higher in PET compared to healthy controls and four were higher in PET compared to both IUGR and healthy controls.

### Placental membrane lipids

**Fig 2:**
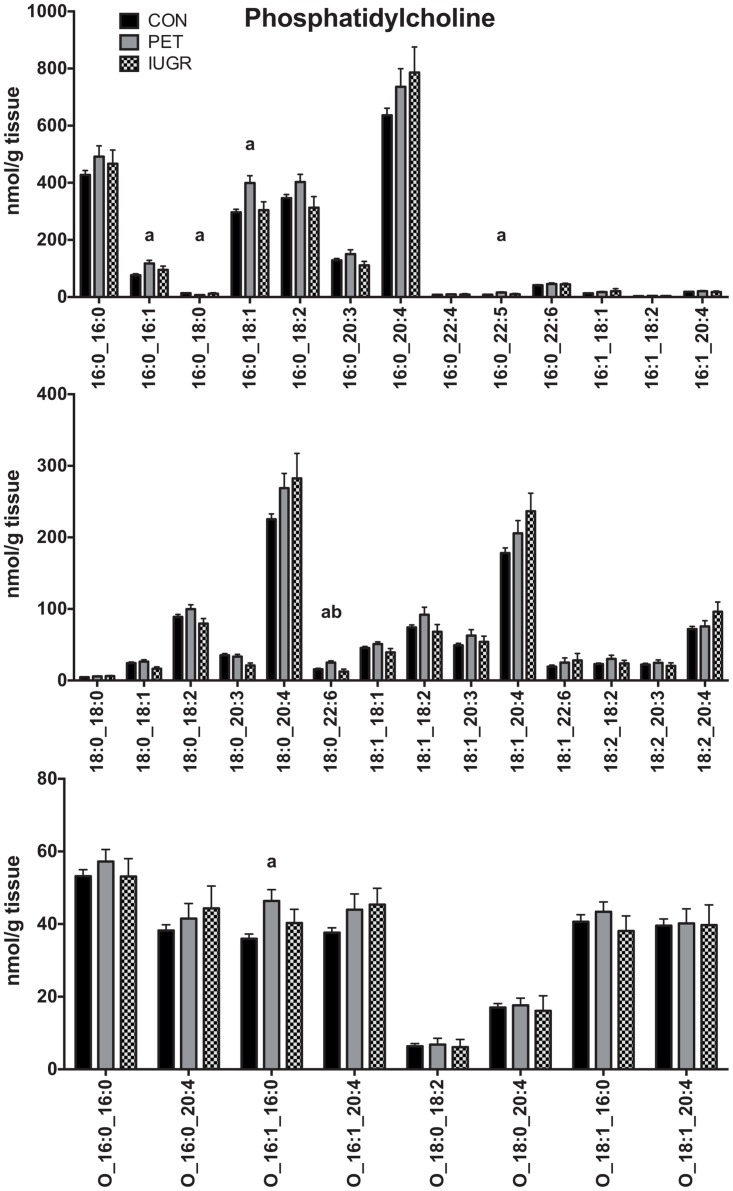
Phosphatidylcholine Lipid Profiles in Placenta. Quantitative comparison of phosphatidylcholine molecular lipid profiles in placenta between control (N = 68), PET (N = 23) and IUGR (N = 10). Values are shown as the mean of all measurements ± SEM. a = p < 0.01 versus control, b = p < 0.01 versus IUGR.

Molecular speciation of the main phospholipids (PC, PE and PS) was compared between groups. A total of 35 molecular species were detected in PC, including 8 ether-linked species (Fig 2). The most abundant PC species contained one 16:0 fatty acids (FA) and either a 16:0, 18:1, 18:2, or 20:4 as the second fatty acid. Long chain PCs were less abundant, and were dominated by PC 18:0_20:4 and PC 18:1_20:4. Of the 35 PC species quantified, five species in PET were at higher concentration relative to healthy controls (16:0_16:1, 16:0_18:1, 16:0_22:5, 18:0_22:6, and O-16:1_16:0) and one species at lower concentration (16:0_18:0), as shown in Fig 2. The only species that were higher in PET compared to both healthy controls and IUGR was PC 18:0_22:6.

**Fig 3:**
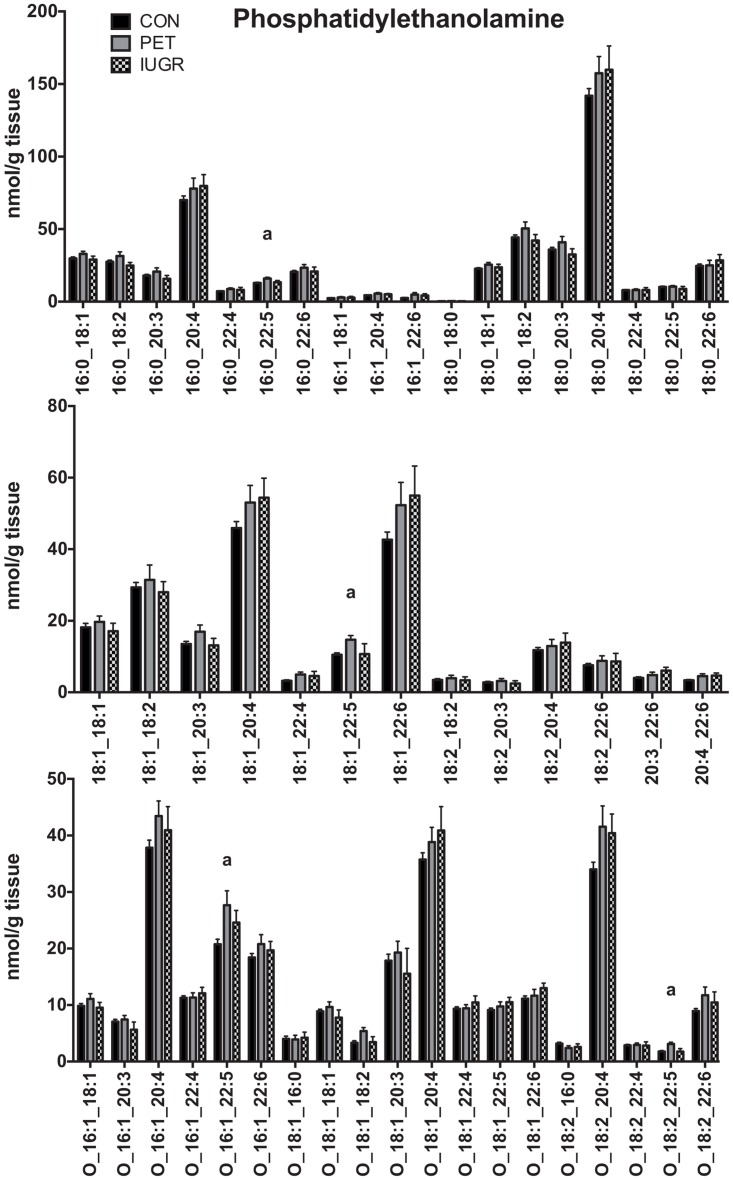
Phosphatidylethanolamine Lipid Profiles in Placenta. Quantitative comparison of phosphatidylethanolamine molecular lipid profiles in placenta between control (N = 68), PET (N = 23) and IUGR (N = 10). Values are shown as the mean of all measurements ± SEM. a = p < 0.01 versus control.

PE is less abundant than PC, representing approximately 10% of total lipid, and a total of 50 molecular species were detected, including 19 ether-linked species (Fig 3). PE is dominated by species containing LC-PUFA, with PE 16:0_20:4 and 18:0_20:4 representing 7% and 15% of total PE respectively. Six species were detected where both attached fatty acids were PUFA, including the very-long chain species PE 20:4_22:5 and PE 20:4_22:6. Of the 50 species detected, four were at higher concentration in placenta compared to healthy controls (16:0_22:5, 18:1_22:5, O-16:1_22:5, O-18:2_22:5). Interestingly, all these increased species contained the 22:5 fatty acid docosapentaenoic acid (DPA).

**Fig 4:**
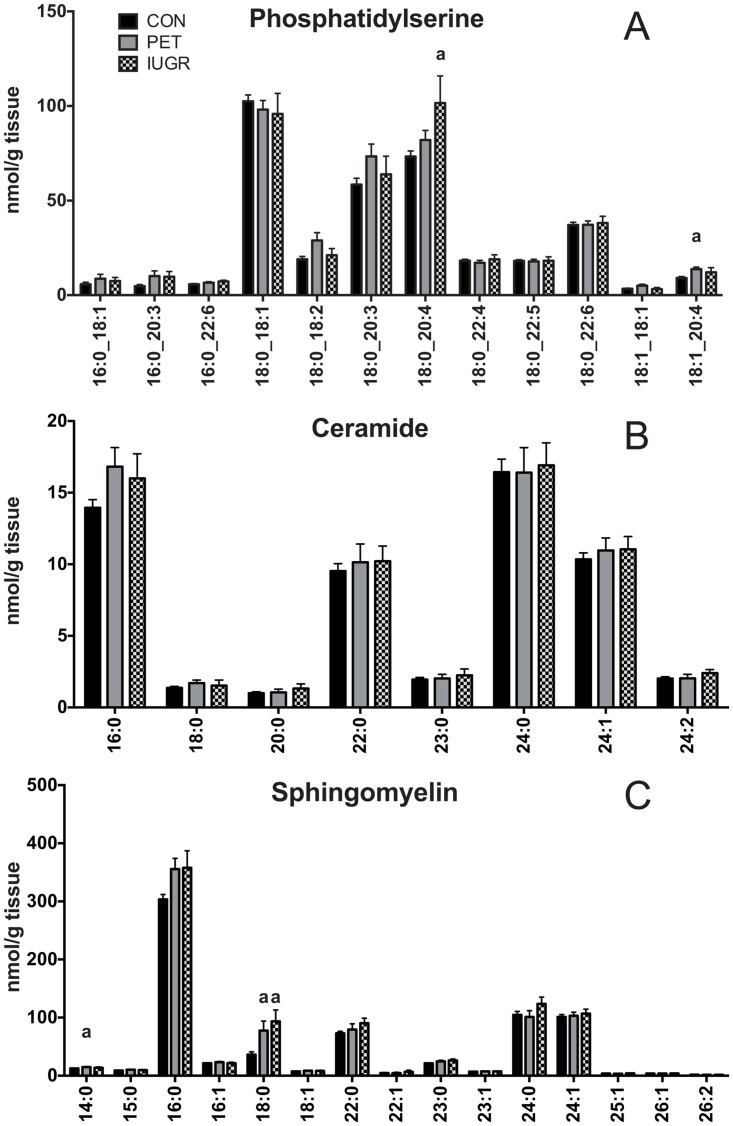
Phosphatidylserine and Sphingolipid Lipid Profiles in Placenta. Lipid Profiles in Placenta. Quantitative comparison of A) phosphatidylserine B) ceramide and C) sphingomyelin molecular lipid profiles in placenta between control (N = 68), PET (N = 23) and IUGR (N = 10). Values are shown as the mean of all measurements ± SEM. a = p < 0.01 versus control.

PS is the least abundant phospholipid in the placenta; approximately 4% of total lipid. Seven of the 12 detected PS species contained an 18:0 FA, six of which also contained a PUFA (Fig 4A). Only two PS species differed between groups, with PS 18:0_20:4 39% higher in IUGR than healthy controls, and PS 18:1_20:4 51% higher in PET than healthy controls.

Ceramide was the lowest abundance lipid class detected in placental tissue, representing under 1% of total lipid. SM represented approximately 8% of total lipid. Eight Cer species ranging from 16:0 to 24:2 were identified, with no differences between groups (Fig 4B). SM had acyl-linked FA chains ranging from 14:0 to 26:2 (Fig 4C). SM 16:0 was the predominant species, comprising 43% of total SM. Other abundant SM species included 18:0, 22:0, 24:0 and 24:1. Of the 15 species of SM detected, two differed between groups, with SM 14:0 20% higher in PET compared to healthy controls while SM 18:0 was higher in both IUGR (157%) and PET (113%) compared to healthy controls.

Placental cholesterol and total PL concentrations were significantly higher (16% and 17% respectively) in PET compared to controls with IUGR having intermediate levels (Table 2). The cholesterol to total PL ratio (an indicator of membrane fluidity) was calculated and was not different among the 3 groups (control 0.57 (0.10); PET 0.57 (0.08); IUGR 0.57 (0.7)).

## Discussion

Presented herein is a comprehensive lipidomics analysis of human placentae with quantitative comparison between placenta from women with healthy pregnancies and those from women with PET. This study has directly ascertained for the first time that placental neutral storage lipid content (TAG and CE) is specifically higher in PET compared to healthy control and IUGR third trimester placenta. These differences remained after correction for gestational age. Others who have not found higher placental TAG content in PET [16] sampled the decidua basalis in an attempt to characterise acute atherosclerosis, a site of cholesterol deposition, rather than sampling the full thickness of the placenta. In the results presented here, lipid analysis was carried out in one random biopsy from each placenta. Sampling location should not have biased our results as it has been shown that FA composition in placenta is homogenous across anatomical locations of the placenta [20].

A recent report of 20 pregnant women (10 with early-onset preeclampsia and 10 healthy pregnancy) does not report similar with the lipid profile nor observe the differences in placenta complicated with PET that we observed [17]. Notably, that report did not incorporate the standard practice of internal standards for normalization of lipid extraction and MS ionization for quantification. Surprisingly, in that report the generally low abundance lipid classes of diacylglyerol and phosphatidylinositol were each reported at over 10% of total lipid signal. Results for the PET samples were even more startling, with over 50% of the total lipid reported as PS, and over 30% as macrolides and lactone polyketides. As mentioned by the authors, macrolides and lactone polyketides are not generally found in mammals. It should be noted that plasma lipidomics were also performed as part of that study, with the reported lipid profile also greatly at odds with the generally accepted composition of human plasma [21].

Unlike PET, placenta complicated with IUGR had normal levels of neutral storage lipids (CE and TAG). This may not be surprising since pregnancies complicated with IUGR are characterised by low maternal mobilisation of fat [22]. Cholesterol and PC were higher in PET and at an intermediate level in IUGR, failing to reach significance perhaps due to low sample size. The differences in PL, the main components of cellular membranes, between PET and controls are broadly similar to previously published data using thin layer chromatography [15]. These alterations in structural lipids may relate to abnormal placental architecture and may be common to both PET and IUGR. PC and cholesterol are key membrane lipids and changes in placental content could be related to the recognised placental pathologies associated with PET and IUGR such as reduced numbers of placental villi and abnormal syncytial membrane structure [23, 24]. Two SM lipid species were higher in PET and IUGR compared to controls in this study. Such enrichment in SM was reported in a lipidomic analysis of human placental syncytiotrophoblast microvesicles from abnormal pregnancies, possibly related to microvesicle biogenesis and inflammatory and oxidative stress [25].

While previously we have provided indirect evidence for ectopic fat accumulation in PET [13], here we provide additional evidence that ectopic fat accumulates in PET placenta. Using direct quantitation we observed a higher concentration of neutral storage lipids (TAG and CE), typical of those found in intra-cellular lipid droplets associated with ectopic fat storage. Our data confirm the previously published observation, using non-quantitative histochemical analysis, of lipid storage droplets in placenta in a rodent model of pre-eclampsia [14] and suggests that ectopic lipid accumulation in placenta is part of the pathology of PET.

In this study we used low-resolution tandem-MS technique for lipid identification and quantification [26]. Lipids were identified by nominal ion mass in the respective precursor-ion or neural-loss scan. Multiple isobaric lipids with different fragmentation profiles are compatible with this workflow as each isobaric lipid is identified in an independent tandem-MS scan. Quantification was achieved by comparison of signal from each target lipid with an internal standard. Each lipid class is represented by at least one internal standard; therefore any inter-class ion suppression is corrected during normalization. However, as only one or two internal standards were utilized per lipid class, the assumption is made that all lipids within the class ionize and fragment with the same efficiency. At lipid concentrations below 100 μM these assumptions hold true for polar lipids [19]. However, TAG quantification may vary across lipid chain length and saturation [27]. To expand these data, additional TAG internal standards would improve quantification by correcting for variation in TAG ionization and fragmentation. In addition, TAG was quantified with neutral-loss scans that are affected by the position of each acyl group on the TAG glycerol backbone. This results in the inability to quantify the fatty acyl composition of each TAG. The application of LC-MS/MS, high-resolution MS/MS or MS^3^ to resolve the TAG molecular lipid species would add valuable insight to the identity of lipids stored in the placenta in PET.

In summary, the data presented here show higher concentrations of TAG and CE in the placentae from PET pregnancies. These data are the first presentation of a comprehensive, quantitative lipidomics analysis of human placenta and provide insight into the pathology of placentae from pregnancy complicated by preeclampsia.

## Supporting Information

S1 File **Table A. Internal standards** Target lipid class, ion detected, internal standard used and amount (nmol) per sample used are shown. **Table B. Target lipid classes and their scan parameters** Target lipid class, ion mode, MS/MS experiment (precursor ion (PI) or neutral loss (NL)), scanning range, and CID energy are shown. **Table C. Fatty acid composition of TAG detected in placental tissue.** The fatty acid species detected in each molecular species of TAG are listed. **Table D. Lipid Molecular Species.** Mean, standard deviation, and ANOVA result for molecular lipid profiles in placenta between control (n = 68), PET (n = 23) and IUGR (n = 10). Units are nmol/g tissue.
(DOCX)

## Abbreviations

CE: cholesteryl ester
Cer: ceramide
GDM: gestational diabetes mellitus
HDL: high density lipoprotein
IUGR: intrauterine growth restriction
LPL: lipoprotein lipase
MS: mass spectrometry
MS/MS: tandem mass spectrometry
MTBE: *tert*-butyl methyl ether
NEFA: non-esterified fatty acid
PC: phosphatidylcholine
PE: phosphatidylethanolamine
PET: preeclampsia
PL: phospholipid
PS: phosphatidylserine
PUFA: polyunsaturated fatty acid
SM: sphingomyelin
TAG: triacylglycerol
