# Supplementary material for: A Lipidomic Analysis of Placenta in Preeclampsia: Evidence for Lipid Storage
Source: PLoS One. 2016 Sep 29;11(9):e0163972. doi: 10.1371/journal.pone.0163972 (PMC5042456; doi:10.1371/journal.pone.0163972)
Supplement: S1 File — Table A. Internal standards Target lipid class, ion detected, internal standard used and amount (nmol) per sample used are shown. Table B. Target lipid classes and their scan parameters Target lipid class, ion mode, MS/MS experiment (precursor ion (PI) or neutral loss (NL)), scanning range, and CID energy are shown. Table C. Fatty acid composition of TAG detected in placental tissue. The fatty acid species detected in each molecular species of TAG are listed. Table D. Lipid Molecular Species. Mean, standard deviation, and ANOVA result for molecular lipid profiles in placenta between control (n = 68), PET (n = 23) and IUGR (n = 10). Units are nmol/g tissue. (DOCX) [file pone.0163972.s001.docx]

**Supporting Information**

**A Lipidomic Analysis of Placenta in Preeclampsia: Evidence for Lipid Storage**

**Simon H.J. Brown^1,2^, Samuel R. Eather^3^, Dilys J. Freeman^4^, Barbara J. Meyer^2,3^ , Todd W. Mitchell^2,3^***

^1^School of Biology, University of Wollongong, Wollongong, NSW, Australia.

^2^Illawarra Health and Medical Research Institute, University of Wollongong, Wollongong, NSW, Australia.

^3^School of Medicine, University of Wollongong, Wollongong, NSW, Australia.

^4^Institute of Cardiovascular and Medical Sciences, University of Glasgow, Glasgow, United Kingdom.

Short Title: **Lipidomics of Placenta in Pre-eclampsia**

*Corresponding author

[toddm@uow.edu.au](mailto:toddm@uow.edu.au) (TWM)

**Table A. Internal standards** Target lipid class, ion detected, internal standard used and amount (nmol) per sample used are shown.

| **Lipid Class** | **Ion** | **Internal Standard** | **nmol/sample** |
| --- | --- | --- | --- |
| PC | [M+H]^+^/ [M+Ac]^-^ | PC 19:0_19:0 | 5 |
| PE | [M+H]^+^/ [M-H]^-^ | PE 17:0_17:0 | 1 |
| SM | [M+H]^+^ | DHSM 12:0 | 5 |
| Cer | [M+H]^+^ | CER 17:0 | 0.25 |
| PS | [M-H]^-^ | PS 17:0_17:0 | 0.5 |
| Cholesterol | [M+NH_4_]^+^ | d_7_ free cholesterol | 20 |
| CE | [M+NH_4_]^+^ | CE 22:1 | 0.5 |
| TAG | [M+NH_4_]^+^ | D_5_ TAG 16:0_16:0_16:0 | 0.1 |

CE, cholesteryl ester; Cer, ceramides; DHSM, dihydrosphingomyelin; PC, phosphatidylcholine; PE, phosphatidylethanolamine; PL, phospholipid; PS, phosphatidylserine; SM, sphingomyelin; TAG, triacylglycerol.

**Table B. Target lipid classes and their scan parameters** Target lipid class, ion mode, MS/MS experiment (precursor ion (PI) or neutral loss (NL)), scanning range, and CID energy are shown.

| **Target Lipid** | **Ion mode** | **MS/MS** | **Range** | **CID energy (eV)** |
| --- | --- | --- | --- | --- |
| PC/SM | + ve | PI 184.1 *m/z* | 640-870 | 40 |
| Cer | + ve | PI 264.4 *m/z* | 400-800 | 35 |
| PE | + ve | NL 141 | 680-830 | 30 |
| PS | + ve | NL 185 | 730-880 | 30 |
| Cholesterol | + ve | NL 35 | 400-420 | 15 |
| CE | + ve | PI 369.4 *m/z* | 610-780 | 25 |
| TAG |  |  |  |  |
| 16:1 | + ve | NL 271.3 | 780-930 | 35 |
| 16:0 | + ve | NL 273.3 | 780-930 | 35 |
| 18:2 | + ve | NL 297.3 | 840-980 | 35 |
| 18:1 | + ve | NL 299.3 | 840-980 | 35 |
| 18:0 | + ve | NL 301.3 | 840-980 | 35 |
| 20:4 | + ve | NL 321.3 | 850-980 | 35 |
| 22:6 | + ve | NL 345.3 | 850-1020 | 35 |
| PL Fatty Acyl |  |  |  |  |
| 16:1 | - ve | PI 253.2 *m/z* | 650-850 | 55 |
| 16:0 | - ve | PI 255.2 *m/z* | 660-880 | 55 |
| 17:0 | - ve | PI 269.3 *m/z* | 600-900 | 55 |
| 18:2 | - ve | PI 279.3 *m/z* | 690-880 | 40 |
| 18:1 | - ve | PI 281.3 *m/z* | 690-900 | 55 |
| 18:0 | - ve | PI 283.3 *m/z* | 690-900 | 55 |
| 19:0 | - ve | PI 297.3 *m/z* | 690-900 | 55 |
| 20:3 | - ve | PI 305.2 *m/z* | 700-900 | 40 |
| 20:4 | - ve | PI 303.2 *m/z* | 700-900 | 40 |
| 22:6 | - ve | PI 327.3 *m/z* | 740-920 | 40 |
| 22:5 | - ve | PI 329.3 *m/z* | 740-920 | 40 |
| 22:4 | - ve | PI 331.3 *m/z* | 740-920 | 40 |

CE, cholesteryl ester; Cer, ceramides; DHSM, dihydrosphingomyelin; PC, phosphatidylcholine; PE, phosphatidylethanolamine; PL, phospholipid; PS, phosphatidylserine; SM, sphingomyelin; TAG, triacylglycerol.

**Table C. Fatty acid composition of TAG detected in placental tissue.** The fatty acid species detected in each molecular species of TAG are listed.

| **TAG Species** | **Fatty acid detected** |
| --- | --- |
| 48:1 | 16:0, 16:1 |
| 48:2 | 16:0, 16:1 |
| 48:3 | 16:1 |
| 50:1 | 16:0, 16:1, 18:0, 18:1 |
| 50:2 | 16:0, 16:1, 18:0, 18:1, 18:2 |
| 50:3 | 16:0, 16:1, 18:1, 18:2 |
| 50:4 | 16:1, 18:2 |
| 52:1 | 16:0, 18:0, 18:1 |
| 52:2 | 16:0, 18:0, 18:1, 18:2 |
| 52:3 | 16:0, 16:1, 18:1, 18:2 |
| 52:4 | 16:0, 16:1, 18:1, 18:2, 20:4 |
| 54:1 | 18:0, 18:1 |
| 54:2 | 18:0, 18:1, 18:2 |
| 54:3 | 18:0, 18:1, 18:2 |
| 54:4 | 16:0, 18:0, 18:1, 18:2, 20:4 |
| 54:5 | 16:0, 18:1, 18:2, 20:4 |
| 56:4 | 18:0, 20:4 |
| 56:5 | 18:0, 18:1, 20:4 |
| 56:7 | 16:0, 18:1, 18:2, 20:4, 22:6 |
| 56:8 | 16:0, 18:2, 20:4, 22:6 |
| 58:8 | 18:0, 18:1, 18:2, 20:4, 22:6 |
| 58:9 | 18:1, 18:2, 20:4, 22:6 |

**Table D. Lipid Molecular Species.** Mean, standard deviation, and ANOVA result for molecular lipid profiles in placenta between control (n=68), PET (n=23) and IUGR (n=10). Units are nmol/g tissue.

|  | CON | | PET | | IUGR | | ANOVA |
| --- | --- | --- | --- | --- | --- | --- | --- |
|  | **Mean** | **SD** | **Mean** | **SD** | **Mean** | **SD** | **P<0.01** |
| CE 14:0 | 2.81 | 1.16 | 4.20 | 2.32 | 2.48 | 1.22 | * |
| CE 15:0 | 1.03 | 1.37 | 1.31 | 1.28 | 0.95 | 0.84 |  |
| CE 16:0 | 28.33 | 9.41 | 36.71 | 12.81 | 24.20 | 7.69 | * |
| CE 16:1 | 14.18 | 5.97 | 20.64 | 7.46 | 13.46 | 5.04 | * |
| CE 18:0 | 1.85 | 1.09 | 3.07 | 2.51 | 2.25 | 1.32 | * |
| CE 18:1 | 63.92 | 24.68 | 98.18 | 42.97 | 62.20 | 23.60 | * |
| CE 18:2 | 135.71 | 50.42 | 157.56 | 60.19 | 111.47 | 48.53 |  |
| CE 18:3 | 6.80 | 2.95 | 9.03 | 3.34 | 6.07 | 2.57 | * |
| CE 20:3 | 8.58 | 5.36 | 16.64 | 15.21 | 7.99 | 5.82 | * |
| CE 20:4 | 34.93 | 11.39 | 45.54 | 17.69 | 33.25 | 10.59 | * |
| CE 22:4 | 1.73 | 1.83 | 4.47 | 5.18 | 2.04 | 1.30 | * |
| CE 22:5 | 3.45 | 2.03 | 5.05 | 2.98 | 3.60 | 2.21 |  |
| CE 22:6 | 8.69 | 4.72 | 12.95 | 7.67 | 7.55 | 3.09 | * |
| Cer 16:0 | 13.95 | 4.65 | 16.82 | 6.43 | 16.01 | 5.41 |  |
| Cer 18:0 | 1.37 | 0.81 | 1.71 | 0.99 | 1.53 | 1.20 |  |
| Cer 20:0 | 0.99 | 0.76 | 1.05 | 1.05 | 1.32 | 1.03 |  |
| Cer 22:0 | 9.53 | 4.26 | 10.14 | 6.14 | 10.21 | 3.36 |  |
| Cer 23:0 | 1.95 | 1.15 | 2.03 | 1.33 | 2.25 | 1.37 |  |
| Cer 24:0 | 16.44 | 7.42 | 16.40 | 8.34 | 16.91 | 4.94 |  |
| Cer 24:1 | 10.35 | 3.71 | 10.96 | 4.16 | 11.04 | 2.86 |  |
| Cer 24:2 | 2.02 | 1.05 | 2.04 | 1.31 | 2.40 | 0.76 |  |
| PC (32:0) 16:0_16:0 | 427.59 | 124.97 | 491.79 | 180.45 | 466.99 | 149.97 |  |
| PC (32:1) 16:0_16:1 | 76.47 | 40.89 | 117.77 | 51.59 | 95.05 | 41.57 | * |
| PC (34:0) 16:0_18:0 | 13.78 | 8.21 | 6.98 | 7.34 | 11.86 | 9.47 | * |
| PC (34:1) 16:0_18:1 | 296.73 | 87.13 | 399.40 | 121.25 | 304.47 | 91.85 | * |
| PC (34:2) 16:0_18:2 | 346.34 | 101.87 | 402.59 | 128.65 | 313.09 | 122.12 |  |
| PC (34:2) 16:1_18:1 | 13.36 | 9.46 | 17.50 | 8.64 | 20.43 | 28.93 |  |
| PC (34:3) 16:1_18:2 | 3.13 | 2.61 | 4.29 | 3.12 | 3.34 | 3.58 |  |
| PC (36:0) 18:0_18:0 | 4.57 | 3.20 | 5.79 | 2.89 | 6.10 | 2.90 |  |
| PC (36:1) 18:0_18:1 | 24.35 | 12.34 | 26.37 | 12.65 | 16.56 | 8.43 |  |
| PC (36:2) 18:0_18:2 | 88.99 | 28.29 | 99.71 | 29.62 | 79.48 | 22.64 |  |
| PC (36:2) 18:1_18:1 | 45.47 | 17.74 | 50.94 | 14.39 | 39.40 | 16.97 |  |
| PC (36:3) 16:0_20:3 | 128.56 | 50.50 | 150.14 | 74.39 | 110.88 | 44.06 |  |
| PC (36:3) 18:1_18:2 | 74.21 | 28.21 | 92.02 | 50.27 | 68.14 | 32.13 |  |
| PC (36:4) 16:0_20:4 | 636.34 | 203.00 | 736.34 | 304.75 | 786.38 | 282.61 |  |
| PC (36:4) 18:2_18:2 | 23.04 | 10.97 | 30.11 | 24.79 | 23.92 | 13.20 |  |
| PC (36:5) 16:1_20:4 | 18.27 | 8.55 | 20.48 | 10.55 | 17.33 | 13.13 |  |
| PC (38:3) 18:0_20:3 | 35.28 | 18.85 | 33.37 | 15.37 | 21.04 | 10.78 |  |
| PC (38:4) 16:0_22:4 | 7.68 | 6.22 | 9.34 | 7.40 | 8.66 | 9.74 |  |
| PC (38:4) 18:0_20:4 | 225.41 | 61.04 | 268.80 | 98.24 | 282.51 | 110.26 |  |
| PC (38:4) 18:1_20:3 | 49.28 | 21.17 | 62.72 | 39.67 | 53.99 | 25.57 |  |
| PC (38:5) 16:0_22:5 | 8.58 | 6.55 | 15.95 | 9.46 | 9.56 | 9.05 | * |
| PC (38:5) 18:1_20:4 | 178.14 | 58.75 | 205.62 | 85.88 | 236.66 | 79.39 |  |
| PC (38:5) 18:2_20:3 | 22.29 | 14.26 | 24.68 | 19.53 | 20.54 | 12.99 |  |
| PC (38:6) 16:0_22:6 | 41.43 | 13.60 | 45.61 | 18.57 | 44.62 | 12.91 |  |
| PC (38:6) 18:2_20:4 | 71.90 | 30.91 | 75.50 | 38.41 | 96.23 | 42.67 |  |
| PC (40:6) 18:0_22:6 | 15.71 | 10.33 | 25.02 | 11.96 | 12.45 | 11.22 | * |
| PC (40:7) 18:1_22:6 | 19.30 | 19.25 | 25.07 | 31.48 | 28.16 | 30.73 |  |
| PC (O-32:0) O-16:0_16:0 | 53.20 | 14.77 | 57.23 | 15.90 | 53.10 | 15.59 |  |
| PC (O-32:1) O-16:1_16:0 | 35.95 | 10.74 | 46.36 | 15.05 | 40.29 | 11.94 | * |
| PC (O-34:1) O-18:1_16:0 | 40.64 | 16.20 | 43.38 | 13.08 | 38.10 | 13.11 |  |
| PC (O-36:2) O-18:0_18:2 | 6.33 | 6.17 | 6.82 | 8.42 | 6.12 | 6.57 |  |
| PC (O-36:4) O-16:0_20:4 | 38.23 | 12.93 | 41.53 | 19.97 | 44.35 | 19.44 |  |
| PC (O-36:5) O-16:1_20:4 | 37.65 | 10.78 | 43.95 | 20.94 | 45.38 | 14.27 |  |
| PC (O-38:4) O-18:0_20:4 | 17.01 | 9.07 | 17.64 | 9.39 | 16.09 | 13.16 |  |
| PC (O-38:5) O-18:1_20:4 | 39.58 | 15.00 | 40.19 | 19.16 | 39.74 | 17.60 |  |
| PE (34:1) 16:0_18:1 | 29.84 | 8.53 | 32.91 | 8.94 | 29.02 | 7.43 |  |
| PE (34:2) 16:0_18:2 | 27.50 | 9.24 | 31.50 | 13.75 | 24.96 | 6.42 |  |
| PE (34:2) 16:1_18:1 | 2.48 | 2.94 | 2.87 | 2.95 | 2.84 | 2.48 |  |
| PE (36:0) 18:0_18:0 | 0.33 | 0.38 | 0.42 | 0.45 | 0.35 | 0.31 |  |
| PE (36:1) 18:0_18:1 | 22.72 | 5.77 | 25.45 | 7.47 | 23.68 | 6.79 |  |
| PE (36:2) 18:0_18:2 | 44.24 | 14.37 | 50.48 | 21.68 | 42.15 | 13.11 |  |
| PE (36:2) 18:1_18:1 | 18.15 | 9.19 | 19.67 | 7.87 | 17.08 | 7.00 |  |
| PE (36:3) 16:0_20:3 | 17.93 | 7.66 | 20.76 | 12.01 | 15.79 | 7.25 |  |
| PE (36:3) 18:1_18:2 | 29.33 | 11.11 | 31.44 | 19.93 | 27.98 | 9.34 |  |
| PE (36:4) 16:0_20:4 | 70.04 | 23.31 | 77.92 | 35.00 | 79.73 | 24.79 |  |
| PE (36:4) 18:2_18:2 | 3.45 | 2.69 | 3.96 | 3.81 | 3.36 | 3.07 |  |
| PE (36:5) 16:1_20:4 | 4.40 | 1.37 | 5.57 | 2.99 | 5.07 | 1.54 |  |
| PE (38:3) 18:0_20:3 | 35.84 | 13.35 | 40.99 | 18.81 | 32.55 | 12.53 |  |
| PE (38:4) 16:0_22:4 | 7.20 | 2.94 | 8.69 | 3.82 | 8.18 | 5.43 |  |
| PE (38:4) 18:0_20:4 | 141.93 | 40.77 | 157.42 | 55.60 | 159.83 | 51.80 |  |
| PE (38:4) 18:1_20:3 | 13.50 | 5.96 | 16.95 | 8.87 | 13.15 | 6.12 |  |
| PE (38:5) 16:0_22:5 | 12.96 | 3.59 | 15.95 | 4.02 | 13.39 | 4.00 | * |
| PE (38:5) 18:1_20:4 | 45.90 | 15.17 | 53.03 | 22.98 | 54.40 | 17.30 |  |
| PE (38:5) 18:2_20:3 | 2.76 | 2.29 | 3.15 | 3.25 | 2.45 | 2.50 |  |
| PE (38:6) 16:0_22:6 | 20.57 | 7.36 | 23.41 | 10.25 | 20.86 | 9.56 |  |
| PE (38:6) 18:2_20:4 | 11.78 | 6.17 | 12.94 | 8.79 | 13.87 | 8.51 |  |
| PE (38:7) 16:1_22:6 | 2.56 | 2.59 | 4.88 | 6.60 | 4.21 | 3.96 |  |
| PE (40:4) 18:0_22:4 | 7.98 | 2.58 | 8.09 | 3.46 | 8.02 | 5.13 |  |
| PE (40:5) 18:0_22:5 | 10.16 | 4.49 | 10.47 | 3.21 | 8.81 | 5.56 |  |
| PE (40:5) 18:1_22:4 | 3.23 | 2.30 | 4.98 | 3.21 | 4.54 | 4.09 |  |
| PE (40:6) 18:0_22:6 | 24.66 | 11.24 | 24.98 | 17.05 | 28.47 | 12.59 |  |
| PE (40:6) 18:1_22:5 | 10.51 | 4.25 | 14.68 | 5.76 | 10.68 | 9.15 | * |
| PE (40:7) 18:1_22:6 | 42.68 | 17.47 | 52.32 | 30.50 | 55.02 | 26.01 |  |
| PE (40:8) 18:2_22:6 | 7.57 | 4.21 | 8.80 | 6.68 | 8.64 | 7.15 |  |
| PE (42:10) 20:4_22:6 | 3.36 | 1.65 | 4.51 | 3.14 | 4.66 | 2.33 |  |
| PE (42:9) 20:3_22:6 | 4.01 | 2.54 | 4.81 | 3.99 | 6.08 | 2.93 |  |
| PE (O-34:1) O-18:1_16:0 | 4.02 | 3.88 | 3.91 | 3.61 | 4.24 | 3.05 |  |
| PE (O-34:2) O-16:1_18:1 | 9.84 | 3.46 | 11.08 | 4.50 | 9.53 | 2.97 |  |
| PE (O-34:2) O-18:2_16:0 | 3.17 | 1.96 | 2.40 | 1.93 | 2.59 | 1.69 |  |
| PE (O-36:2) O-18:1_18:1 | 8.90 | 3.02 | 9.66 | 4.38 | 7.78 | 4.27 |  |
| PE (O-36:3) O-18:1_18:2 | 3.33 | 2.91 | 5.39 | 2.99 | 3.44 | 2.99 |  |
| PE (O-36:4) O-16:1_20:3 | 7.07 | 3.17 | 7.45 | 3.39 | 5.64 | 4.22 |  |
| PE (O-36:5) O-16:1_20:4 | 37.84 | 11.10 | 43.43 | 12.81 | 40.94 | 13.18 |  |
| PE (O-38:4) O-18:1_20:3 | 17.85 | 9.46 | 19.27 | 9.62 | 15.55 | 14.18 |  |
| PE (O-38:5) O-16:1_22:4 | 11.27 | 3.07 | 11.33 | 4.03 | 12.08 | 3.36 |  |
| PE (O-38:5) O-18:1_20:4 | 35.74 | 9.84 | 38.85 | 12.44 | 40.89 | 13.31 |  |
| PE (O-38:6) O-16:1_22:5 | 20.76 | 7.22 | 27.66 | 12.30 | 24.59 | 6.83 | * |
| PE (O-38:6) O-18:2_20:4 | 34.00 | 10.55 | 41.55 | 17.60 | 40.41 | 10.73 |  |
| PE (O-38:7) O-16:1_22:6 | 18.42 | 5.82 | 20.80 | 7.97 | 19.69 | 5.01 |  |
| PE (O-40:5) O-18:1_22:4 | 9.35 | 2.80 | 9.42 | 3.15 | 10.47 | 3.58 |  |
| PE (O-40:6) O-18:1_22:5 | 9.12 | 2.97 | 9.76 | 3.89 | 10.53 | 2.61 |  |
| PE (O-40:6) O-18:2_22:4 | 2.86 | 1.47 | 2.93 | 1.64 | 2.81 | 2.14 |  |
| PE (O-40:7) O-18:1_22:6 | 11.13 | 3.95 | 11.67 | 5.29 | 13.00 | 2.82 |  |
| PE (O-40:7) O-18:2_22:5 | 1.78 | 1.27 | 3.11 | 1.48 | 1.77 | 1.69 | * |
| PE (O-40:8) O-18:2_22:6 | 8.94 | 3.60 | 11.72 | 7.00 | 10.46 | 5.93 |  |
| PS (34:1) 16:0_18:1 | 5.75 | 8.72 | 8.68 | 11.32 | 7.49 | 5.94 |  |
| PS (36:1) 18:0_18:1 | 102.48 | 27.65 | 98.11 | 22.97 | 95.86 | 34.22 |  |
| PS (36:2) 18:0_18:2 | 19.01 | 12.14 | 28.94 | 19.73 | 21.16 | 11.04 |  |
| PS (36:2) 18:1_18:1 | 3.41 | 2.46 | 5.03 | 3.52 | 3.16 | 2.80 |  |
| PS (36:3) 16:0_20:3 | 4.76 | 6.77 | 10.13 | 13.13 | 9.77 | 8.87 |  |
| PS (38:3) 18:0_20:3 | 58.42 | 27.69 | 73.36 | 31.31 | 63.85 | 30.39 |  |
| PS (38:4) 18:0_20:4 | 73.30 | 24.48 | 82.01 | 24.54 | 101.58 | 45.49 | * |
| PS (38:5) 18:1_20:4 | 9.13 | 5.97 | 13.76 | 5.10 | 12.13 | 7.60 | * |
| PS (38:6) 16:0_22:6 | 5.75 | 2.98 | 6.58 | 2.43 | 7.27 | 1.97 |  |
| PS (40:4) 18:0_22:4 | 18.25 | 5.88 | 17.15 | 6.18 | 18.97 | 7.50 |  |
| PS (40:5) 18:0_22:5 | 18.14 | 5.29 | 17.86 | 5.31 | 18.21 | 6.45 |  |
| PS (40:6) 18:0_22:6 | 37.14 | 11.72 | 37.21 | 9.65 | 38.18 | 11.26 |  |
| SM 14:0 | 12.51 | 2.76 | 14.95 | 4.06 | 13.72 | 4.60 | * |
| SM 15:0 | 8.99 | 1.99 | 10.37 | 2.43 | 9.70 | 3.05 |  |
| SM 16:0 | 303.48 | 68.31 | 355.66 | 88.34 | 357.74 | 92.92 |  |
| SM 16:1 | 21.55 | 5.22 | 23.19 | 6.24 | 21.58 | 5.39 |  |
| SM 18:0 | 36.41 | 39.67 | 77.64 | 78.67 | 93.60 | 61.61 | * |
| SM 18:1 | 7.53 | 2.78 | 8.63 | 3.95 | 8.27 | 3.28 |  |
| SM 22:0 | 73.08 | 28.01 | 79.57 | 46.63 | 90.56 | 26.60 |  |
| SM 22:1 | 4.75 | 7.75 | 4.41 | 6.35 | 6.59 | 9.09 |  |
| SM 23:0 | 21.30 | 5.91 | 24.75 | 8.37 | 26.27 | 7.66 |  |
| SM 23:1 | 6.96 | 2.08 | 7.50 | 1.94 | 7.49 | 2.26 |  |
| SM 24:0 | 104.83 | 48.46 | 101.18 | 51.73 | 123.59 | 36.83 |  |
| SM 24:1 | 101.14 | 33.40 | 103.17 | 29.11 | 106.95 | 24.11 |  |
| SM 25:1 | 3.75 | 1.61 | 3.42 | 1.53 | 4.17 | 2.09 |  |
| SM 26:1 | 3.62 | 1.40 | 3.80 | 1.72 | 3.90 | 1.27 |  |
| SM 26:2 | 1.68 | 1.20 | 1.60 | 1.31 | 1.57 | 0.95 |  |
| TAG 48:1 | 4.11 | 2.46 | 6.36 | 3.37 | 3.48 | 1.32 | * |
| TAG 48:2 | 1.78 | 1.21 | 2.58 | 1.35 | 1.52 | 0.81 |  |
| TAG 48:3 | 0.26 | 0.22 | 0.38 | 0.22 | 0.31 | 0.18 |  |
| TAG 50:1 | 20.72 | 11.77 | 29.80 | 14.36 | 15.03 | 5.30 | * |
| TAG 50:2 | 19.99 | 11.64 | 29.45 | 13.67 | 15.06 | 4.34 | * |
| TAG 50:3 | 6.21 | 3.85 | 8.63 | 4.02 | 4.65 | 1.40 |  |
| TAG 50:4 | 0.80 | 0.50 | 1.14 | 0.52 | 0.73 | 0.27 |  |
| TAG 52:1 | 6.89 | 4.46 | 9.41 | 6.42 | 4.62 | 1.87 |  |
| TAG 52:2 | 39.30 | 23.41 | 49.17 | 22.16 | 26.24 | 10.13 |  |
| TAG 52:3 | 33.45 | 19.70 | 41.94 | 19.01 | 22.60 | 10.25 |  |
| TAG 52:4 | 10.73 | 6.50 | 14.35 | 7.62 | 7.83 | 3.82 |  |
| TAG 54:1 | 0.92 | 0.87 | 1.25 | 0.93 | 0.70 | 0.27 |  |
| TAG 54:2 | 3.51 | 2.15 | 5.22 | 3.25 | 2.49 | 0.90 | * |
| TAG 54:3 | 9.57 | 5.61 | 13.60 | 7.25 | 6.98 | 3.19 | * |
| TAG 54:4 | 11.23 | 6.50 | 17.38 | 9.37 | 9.21 | 3.85 | * |
| TAG 54:5 | 8.19 | 5.14 | 13.55 | 7.69 | 7.53 | 3.21 | * |
| TAG 56:4 | 1.19 | 0.77 | 1.99 | 1.10 | 1.15 | 0.62 | * |
| TAG 56:5 | 3.45 | 2.16 | 5.55 | 2.70 | 3.47 | 1.27 | * |
| TAG 56:7 | 5.05 | 3.80 | 8.12 | 3.82 | 5.42 | 2.43 | * |
| TAG 56:8 | 2.75 | 1.95 | 4.61 | 2.55 | 3.23 | 1.77 | * |
| TAG 58:8 | 2.80 | 2.53 | 4.57 | 2.23 | 3.30 | 1.82 | * |
| TAG 58:9 | 2.24 | 2.11 | 3.76 | 1.85 | 2.90 | 1.64 | * |
